# Enhanced oral bioavailability and bioefficacy of phloretin using mixed polymeric modified self‐nanoemulsions

**DOI:** 10.1002/fsn3.1637

**Published:** 2020-05-28

**Authors:** Yiling Wang, Dongli Li, Huiqiong Lin, Sen Jiang, Lei Han, Shuli Hou, Shuying Lin, Zhefeng Cheng, Wangqing Bian, Xinxin Zhang, Yan He, Kun Zhang

**Affiliations:** ^1^ School of Biotechnology and Health Science Wuyi University Jiangmen China; ^2^ School of Biomedical and Pharmaceutical Sciences Guangdong University of Technology Guangzhou China; ^3^ Shanghai Institute of Materia Medica Chinese Academy of Sciences Shanghai China

**Keywords:** anti‐inflammatory, bioavailability, pharmacokinetics, phloretin, self‐nanoemulsions, solubility

## Abstract

Phloretin (Ph) is a natural active ingredient with wide biological properties. However, its poor water‐solubility and low oral bioavailability limit the application significantly in functional food and drug. This study was to explore the mixed polymer Pluronic® F127 and P123 modified the different triglycerides (LCT, MCT, SCT) in self‐nanoemulsions (SNEs) for enhancing the oral bioavailability and bioefficacy of Ph. The SNEs were characterized in terms of physical property study, lipolysis study, pharmacokinetic study, and anti‐inflammatory effect. The water‐solubility of LCT‐Ph‐SNE increased 3000‐fold compared with Ph solution. Pharmacokinetic study of SNEs and other carriers (HP‐β‐CD, PVP) results indicated that LCT‐Ph‐SNE was 7.9‐fold more bioavailable compared with unformulated Ph. The anti‐inflammatory activity of LCT‐Ph‐SNE in vivo represented a 6.8‐fold enhancement compared with unformulated Ph. This novel SNE formulation may also be used for other poorly soluble ingredients with high loading capacity, which made a significant impact on functional food and drug.

## Graphical abstract

1. A novel self‐nanoemulsions modified by mixed polymers are effective in enhancing the bioavailability of phloretin (Ph).
2. The mixed polymer modified self‐nanoemulsions (SNEs) to minimize the degradation clearance by enzymes in mucus.
3. Enhance the oral bioavailability and bioefficacy of poorly soluble ingredients phloretin (Ph).
4. The water‐solubility and oral bioavailability of LCT‐Ph‐SNE has significantly increased.
5. The anti‐inflammatory activity of LCT‐Ph‐SNE in vivo is much higher that the unformulated Ph.

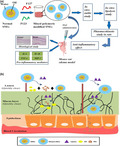

## 1 INTRODUCTION

Phloretin (2',4',6'‐trihydroxy‐3‐(4‐hydroxyphenyl)‐propiophenone, Ph, Figure 2b) is an important natural dihydrochalcone existing in apple, pear, and other juicy fruits with wide biological properties such as anti‐inflammatory, antioxidant, anticancer, anti‐cardiovascular disease, and anti‐aging (Bungaruang, Gutmann, & Nidetzky, 2016; Hardie, 2015). However, the solubility of Ph in water is only about 0.2 mM, which significantly limits its applications in medicine and functional food (Crespy et al., 2001). More importantly, reports revealed that a large amount of orally administered Ph is excreted in the urine and the feces. The low bioavailability of Ph is due to the poor oral absorption, rapid metabolism, and clearance from the human body (Monge, Solheim, & Scheline, 1984).

In the past, there were several kinds of technologies to improve the dissolution, such as solid dispersion technique with Polyvinylpyrrolidone (PVP) or simple co‐evaporation method with hydroxypropyl‐β‐cyclodextrin (HP‐β‐CD), but it was still unable to improve the solubility of phloretin effectively (Vo, Park, & Lee, 2013; Wei, Zhang, Memon, & Liang, 2017). And in recent years, the self‐nanoemulsions (SNEs) have attracted increasing interest in food and drug research, due to its excellent ability to improve the solubility of hydrophobic drugs. It was widely used as a significant source of energy, essential fatty acids (FAs), and fat‐soluble vitamins, which is a beneficial clinical effect for the terminally ill, pediatric, and long‐term parenteral nutrition patients. Though the nanoemulsions were regarded to be a considerable carrier for the poorly water‐soluble drugs, nevertheless it still has some potentially limiting factors that must be overcome. It was likely to be degraded in gastrointestinal (GIT), leading to prompt release and precipitation of encapsulated drugs, which removed by intestinal mucus, decreasing the delivery capability of SNEs. So the traditional SNEs need the further improvement, enabling them to be resistant to luminal digestion and capable of traversing the mucus layer rapidly. People have studied its stability about the oil phase, but few people were aware that the emulsifiers would significantly influence the drug's absorption, distribution, metabolism, and excretion in the body. Therefore, we need to search for an efficient self‐nanoemulsion system for the hydrophobic compound (phloretin) to improve its bioavailability and bioefficacy.

In previous papers, it reported that the nanoemulsion with the F127 coating developed to minimize the degradation clearance by enzymes in mucus (Song et al., 2018; Yu et al., 2018). In this study, through the effect of the characteristics on in vitro and in vivo stabilities, we found that the effectiveness of SNEs with F127/P123 mixture coating at inhibiting drug digestion was strongly correlated to the physicochemical factors of the nanoemulsions. The physicochemical factors are including carbon chain length, steric hindrance, surface tension, concentration, charge, structure, lipid digestion, and activity of emulsions. Therefore, for more effective drug treatment, we not only study the function of the physical and chemical properties of Nanoemulsion, but also the function of how the rats body's response to the administration of water‐soluble drugs. By measuring the changes in physical and chemical properties of different SNEs, we could have a better understanding of its interfacial behavior at oil–water interfaces. It may be altered after nanoemulsion formation using some approaches. The Ph solution and suspension (PVP‐Ph) was chosen as the reference formulation. An in vitro digestion model was used to investigate the effect of lipid composition and type of formulation on drug solubilization and lipid digestion. The bioavailability of the drug from the tested formulations was investigated in rats. Finally, the comparison of anti‐inflammatory effect to further prove that developed SNE formulations could improve drug solubility and oral bioavailability.

**FIGURE 1:**
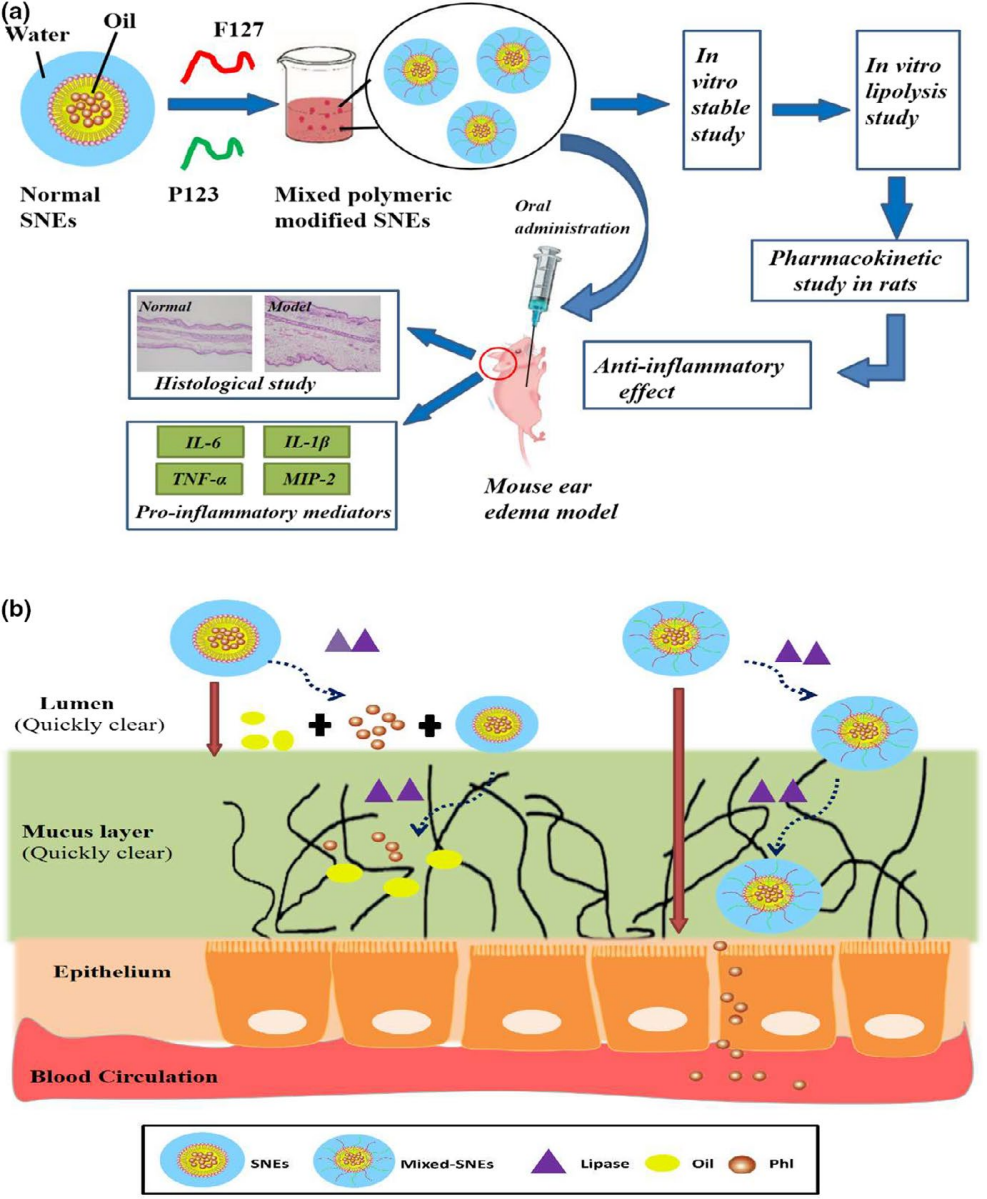
A schematic diagram for (a) the design of SNEs and its bioefficacy study, (b) the transport process of the normal SNEs and Mixed‐SNEs (F127/ P123 mixed polymer modified self‐nanoemulsions) through the mucus layer and epithelial barrier

The purpose of this study was to develop SNE formulations intended to enhance the solubility and oral bioavailability of Ph and to compare the performance of different formulations in pharmacokinetic study. The oils were rapidly surrounded by surfactants, which were adsorbed to droplet surface lead to decreasing the interfacial tension; thus, we could found that the F127/P123 polymeric mixture had highly surface active (Figure 1). The limited permeability of particles through mucus can further lead to their clearance from the GIT, resulting in poor absorption (Yu et al., 2018). It has been reported that nanoparticles (NPs) modified with polymers, which has a long hydrophilic chain, such as Pluronic® F127, P123 or polyethylene glycol (PEG) polymers, could exhibit excellent mucus diffusion ability. Therefore, we hypothesize that SNEs will efficiently overcome both lipases and mucus barriers if they are decorated with hydrophilic polymers. In this study, the optimized SNE with improved physicochemical characteristics provides a more potent formulation of Ph as a therapeutic and functional food ingredient. Moreover, this work has important implications for the pharmaceutical food by using the optimized nanoemulsion systems.

**FIGURE 2:**
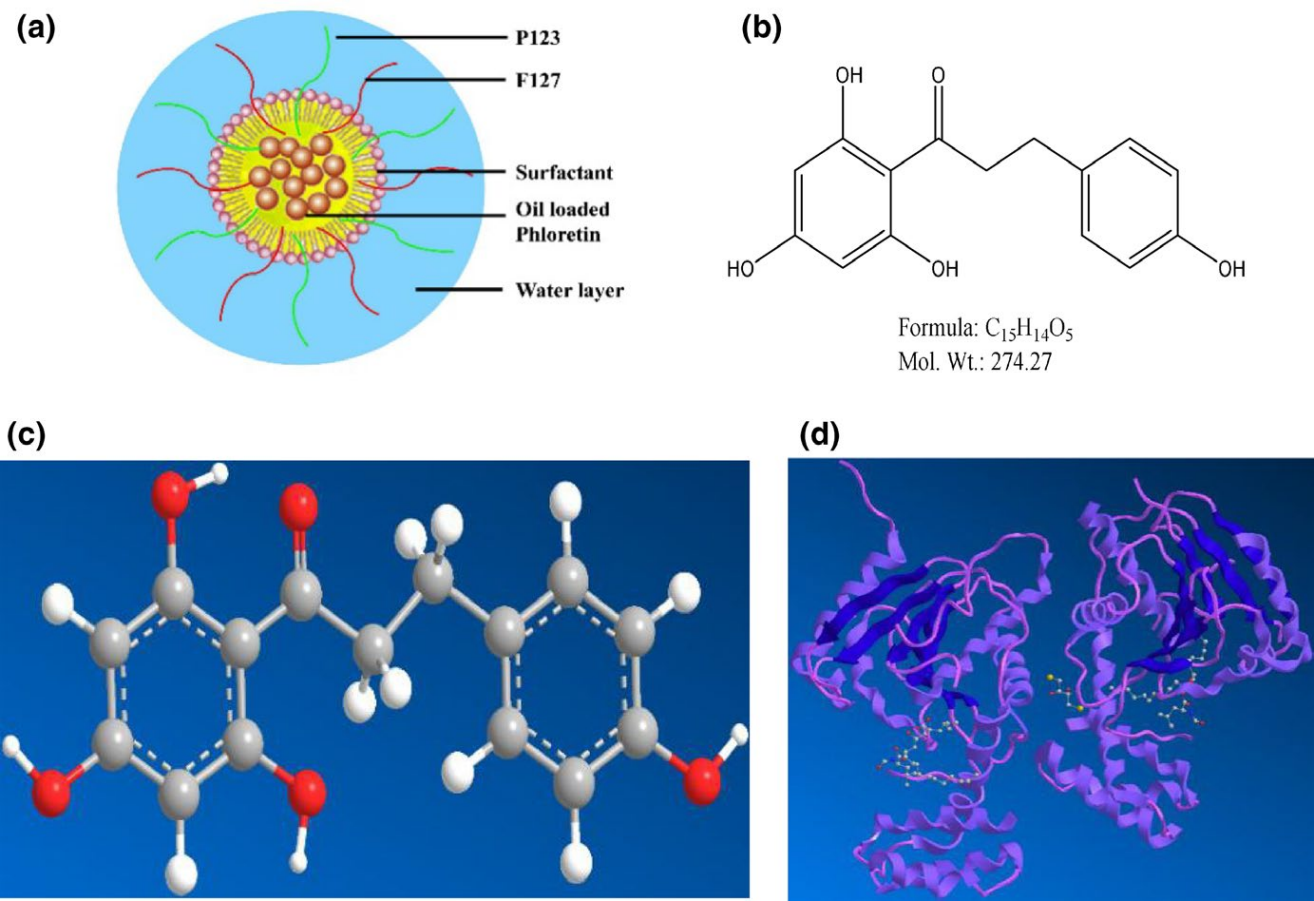
Nanoemulsion characterization. (a) schematic diagram of Ph‐SNEs; (b) chemical structure of Ph; (c) 3D chemical structure of Ph; (d) the interactional model between SNEs and pancreatic lipase was established using AutoDock software

## 2 MATERIALS AND METHODS

### 2.1 Materials

Ph (purity > 98%) was purchased from Xi'an Tongze Biotechnology Co., Ltd. (Xi'an, China). Glyceryl tributyrate (SCT) was purchased from Wuhan far Cheng co‐Creation Technology Co., Ltd. (Wuhan, China). Medium‐chain triglycerides (MCT) was purchased from Asia botanicals Sdn. Bhd. (Selangor, Malaysia). MAISINE® 35–1 (LCT) was donated by Gattefosse S.A (Lyon, France). Carbomer 940, Cremophor EL, and Pluronic P123 were purchased from BASF AG (Ludwigshafen, Germany). Pluronic® F127 was supplied by Sigma‐Aldrich, USA. Ethanol, Propylene glycol, and Porcine pancreatic lipase (1:300) were purchased from Shanghai Aladdin Biochemical Technology Co., Ltd. (Shanghai, China). IL‐6, IL‐1β, TNF‐α, and MIP‐2 were purchased from Jiangsu Enzyme‐Linked Biotechnology Co., Ltd. (Jiangsu, China). BCA Protein Quantification Kit was purchased from Beyotime Biotechnology Co., Ltd. (Shanghai, China). Acetonitrile of high‐performance liquid chromatography (HPLC) grade was obtained from Mreda Technologyinc Inc (USA), and other chemicals were of analytical grade.

### 2.2 Preparation of the Ph‐loaded self‐nanoemulsions (Ph‐SNEs)

Compositions of the Ph‐SNEs are given in Table 1. Each SNE consists of ethanol, 1,2‐propanediol, oil phase, and surfactant, (1:1: 4:6, w/w). Briefly, Pluronic P123 (25 mg) and Pluronic F127 (25 mg) was firstly dissolved in the mixture of co‐solvents (50 mg ethanol and 50 mg 1,2‐propanediol). Ph (400 mg) was dissolved in the mixture of oil phase (200 mg, SCT, MCT or LCT) and surfactant (200 mg, Cremophor EL) under stirring for 20 min at 50°C. The hot oil phase was dispersed in the water phase (the co‐solvents of the ethanol, 1,2‐propanediol) at the same temperature. These Ph‐SNEs were prepared using emulsification followed by vibrating for 20 min at 50°C to make it homogeneity. The final concentration of Ph in the nanoemulsion was 275 mg/g.

**TABLE 1:** Compositions of the Ph‐SNEs

Sample	Compositions
SCT‐Ph‐SNE	Phloretin, Ethanol, 1,2‐propanediol, Glyceryl tributyrate (SCT), Cremophor EL, Pluronic® P123, Pluronic® F127
MCT‐Ph‐SNE	Phloretin, Ethanol, 1,2‐propanediol, Medium‐chain triglycerides (MCT), Cremophor EL, Pluronic® P123, Pluronic® F127
LCT‐Ph‐SNE	Phloretin, Ethanol, 1,2‐propanediol, MAISINE® 35‐1 （LCT）, Cremophor EL, Pluronic® P123, Pluronic® F127

### 2.3 Characteristics of SNEs

#### 2.3.1 Droplet size, size distribution, and ζ‐potential

The prepared nanoemulsions were diluted to 200 times with deionized water and mixed well. The particle size, PDI, and zeta potential were measured by the dynamic light scattering method (Nano ZS, Malvern Instruments Ltd, UK). The light source was at a fixed scattering angle of 90° at 25°C, with 633 nm and 30 mW power (Fan et al., 2011; Huang, Wu, Tu, Lai, & Liou, 2015). Meantime, to the evaluation of the storage stability, the samples were analyzed by mean droplet sizes with Zetasizer Nano after storage during storage (30 days) without light at room temperature (Yuting, Yuzhu, Wally, & Jiang, 2017).

#### 2.3.2 TEM morphology analysis

The morphology was observed by transmission electron microscopic (TEM) (HT7700, Hitachi, Tokyo, Japan). Then, it was diluted with 100 ml of deionized water and mixed well. The 10 μl of diluted nanoemulsions was stained with 1% phosphotungstic acid (PTA) for 1 min and then dropped on a copper grid for 2–3 min. The specimens were transferred to the TEM and analyzed at 100 kV after drying in air at room temperature.

### 2.4 Physical properties of emulsions

#### 2.4.1 Solubility study

To determine solubility, an excess amount of sample was added into 10 ml distilled water. The samples were placed in a shaker and shaken for 24 hr at room temperature to attain equilibrium mixture. The mixture was centrifuged at 10,000 rpm for 10 min and excess insoluble Ph was filtrated through the microporous membrane (Azzi, Jraij, Auezova, Fourmentin, & Greige‐Gerges, 2018). Then, the 20 μl filtrated sample was subjected to HPLC analysis described in 2.7. Pharmacokinetic study.

#### 2.4.2 Optical determination of interfacial tension

The interfacial tensions were measured at the air‐water interface of different nanoemulsions, using the pendant drop technique by a fully automatic contact angle analyzer (OCA100, Dataphysics, Germany). An inverted nanoemulsion drop was formed at the tip of the needle fitted to a syringe with a total volume of 100 μl. The continuous droplets pushed out from the tip of needle between 30 and 10 μl. The video images of drop shape were captured by camera until the nanoemulsion drop detached from the tip of the needle due to the decreased interfacial tension. The drop shape in each image was analyzed by the contour analysis system (SCA22 software), based on the Young‐Laplace evaluation of pendant drops (Donsi, Senatore, Huang, & Ferrari, 2010; Shu et al., 2018; Tran, Guo, Song, Bruno, & Lu, 2014).

#### 2.4.3 Apparent viscosity

Viscosity measurements (Pa·s) were performed by using a Vibro Viscometer (SV‐10, A&D Company, Tokyo, Japan) vibrating at 30 Hz, with constant amplitude and working at room temperature. Aliquots of 10 ml of each nanoemulsion were used for determinations. During shear rate from 0 to 100 s^−1^, apparent viscosity (Pa·s) was recorded (Lu, Xiao, & Huang, 2018).

#### 2.4.4 In vitro stability study

The in vitro stability study was measured by monitoring the particle size and PDI of SNEs in PBS (pH 7.4) and biorelevant media at 37°C for 24 hr using a water‐bath shaker. The biorelevant media included simulated gastric fluid (SGF) and simulated intestinal fluid (SIF). All the media were prepared as previously reported (Huang et al., 2015; Wilde, Garcia‐Llatas, Lagarda, Haslam, & Grundy, 2019). The SGF was composed of 0.2% sodium chloride (NaCl) (w/v) and 0.32% pepsin (w/v); then, the pH was adjusted to 1.2 by 0.1 M HCl. The SIF was composed of 0.68% monobasic potassium phosphate (KH_2_PO_4_) (w/v) and 1% pancreatin (w/v); then, the pH was adjusted to 6.8 by 0.1 M NaOH solution.

### 2.5 In vitro lipolysis study

The lipolysis study was performed by an in vitro lipid digestion model as previously describe (Wilde et al., 2019). Briefly, 100 mg SNE was dispersed in 19 ml 50 mM Tri‐maleate digestion buffer (pH 7.5). Digestion study started by adding 1 ml 10% (w/v) porcine pancreatic lipase solution (4,000 tributyrin units/ml). To neutralize the fatty acid produced by lipolysis, the pH of the mixture was maintained at 7.5 ± 0.05 by adding NaOH solution (1 M) manually. During the digestion period, the mixture was agitated by using a magnetic stirring apparatus at 37 ± 0.5°C. The reaction was stopped when the pH could not drop below 7.45 units for 30 min.

### 2.6 Pharmacokinetic study in rats

The pharmacokinetic study was investigated in Sprague Dawley rats (300–320 g) purchased from the Guangdong Medical Laboratory Animal Center (Guangzhou, China) and the approval document is SCXK/2018‐0002. The rates were kept for 7 days in the environmentally controlled room (12/12 hr dark/light cycle, 25 ± 2°C, 50% humidity) before the experiments study for acclimatization to animal house conditions. All rats were given free access to diet and water. All animal experimental protocol used in this study was approved by the Guangdong Medical Laboratory Animal Center and performed in compliance with the relevant laws and institutional guidelines.

The rats were randomly divided into six groups (*n* = 5) and fasted for 12 hr before drug administration. A single dose of 400 mg/kg Ph solution, SCT‐Ph‐SNE, MCT‐Ph‐SNE, and LCT‐Ph‐SNE dissolved in 5% PEG400 solution was oral administration, respectively. The 0.4 ml of blood was taken from the retro‐orbital plexus under isoflurane anesthesia and then collected at predetermined intervals (5 min predose, 0.083, 0.25, 0.5, 1, 2, 4, 6, 8, and 12 hr postdose) after oral administration. The blood samples were centrifuged at 5,000 rpm for 10 min to separate plasma. The plasma samples were stored at −20°C until analyzed. After treatment, the Ph concentration of the plasma sample was measured by HPLC.

Before analyzing, plasma was thawed at 37°C. 0.2 ml sample was added to 0.2 ml acetonitrile and vortexed for 1 min. The mixture was centrifuged at 12,000 rpm for 5 min, and the upper phase was collected. Then, 20 μl sample was injected into HPLC for analysis on Ph concentration of plasma.

All HPLC analyses were performed on a Shimadzu LC‐20A HPLC with DIKMA C18 Reversed‐phase column (4.6 mm × 200 mm, 5 μm). The column temperature was maintained at 30°C, and the mobile phase was 30% acetonitrile in water with a delivery flow rate of 1 ml/min. PDA detector (detection wavelength 286 nm) was used. The pharmacokinetic parameters were calculated by the pharmacokinetic software WinNonlin Standard Edition v1.1 (Pharsight Corp., Mountain View, CA, USA). The Ph, Blank plasma + Ph, and plasma sample at 0.083h in rats were analyzed with HPLC based on the above chromatographic conditions. As shown in the Figure S1, the retention time of Ph in HPLC is approximately 14.2 min with a good separation to plasma constituents. The regression equation for Ph was *y* = 14885*x* + 2,780.3 (*r* = 0.9994), which was a good linear relationship between drug concentration and peak area. The precision was measured at six different concentrations (0.5, 1.0, 2.0, 5.0, 10.0, 20.0 μg·ml‐1). The percent relative standard deviation(RSD%) value is less than 2%.The mean extraction recovery was 82.02 ± 1.2%, 84.67 ± 1.5% and 81.51 ± 0.85% at 0.5, 10.0, 20.0 μg·ml‐1, respectively. The limit of quantization (S/*N* = 10) was estimated to be 0.3 μg/ml, and the limit of detection (S/*N* = 3) of phloretin was estimated to be 0.1 μg/ml in plasma. It indicated good accuracy and precision of the developed method.

### 2.7 Anti‐inflammatory effect

The anti‐inflammatory effect was carried out on the 12‐O‐tetradecanoyl phorbol‐13‐acetate (TPA)‐induced mouse ear edema model. The male BALB/c rats (20–25 g) were purchased from the Guangdong Medical Laboratory Animal Center (Guangzhou, China), and the approval document is SCXK/2018‐0002. The animals were kept for 7 days in the environmentally controlled room (12/12 hr dark/light cycle, 25 ± 2°C, 50% humidity) before the experiments study for acclimatization to animal house conditions. All rats were given free access to diet and water. All animal experimental protocol used in this study was approved by the Guangdong Medical Laboratory Animal Center and performed in compliance with the relevant laws and institutional guidelines.

The BALB/c rats were randomly divided into the control group, TPA model group, Ph group and SNEs group (*n* = 5). Rats in each group were orally administered the Ph solution (suspended in 5% PEG400), SCT‐Ph‐SNE, MCT‐Ph‐SNE, and LCT‐Ph‐SNE (200 mg·Kg^−1^), respectively. After one hour, both rat ears of the TPA model group, Ph group, and SNEs group were topically treated with TPA (0.008 nM in acetone), while the rat ears of the control group were topically treated with acetone. Then, the rats were killedafter 6 hr. Finally, ear punches of 6 mm diameter from rat ears were taken and weighed. The inhibitory effects (IE) = [(TPA alone) − (test compound plus TPA)]/[(TPA alone) − (acetone alone)] × 100%.

Rat ears were removed in toto, fixed in 10% formalin, decalcified in EDTA buffer, subjected to a series progression of dehydration, and then embedded in paraffin. Samples were serially sectioned into 4 μm and processed routinely for hematoxylin and eosin (H&E) staining. The histological changes were observed under a microscope.

The IL‐6, IL‐1β, TNF‐α, and MIP‐2 concentrations were determined by the enzyme‐linked immunosorbent assay (ELISA) according to the manufacturer's instructions. The results of cytokine determination were corrected by the amount of tissue protein. The protein content of tissues was determined by the Bradford method using the Protein Quantification Kit.

### 2.8 Statistical analysis

All data were presented as mean ± standard deviation (STD). Data were compared by independent Student's *t* test was used to compare the means of two groups. The level of significance was set at *p* < .05 and *p* < .01, which was considered as significant and highly significant. All the statistical analysis was done by using SPSS software (SPSS, Inc., Chicago, IL, USA, version 17.0).

## 3 RESULTS AND DISCUSSION

### 3.1 Characterization of SNEs

#### 3.1.1 Droplet size, size distribution, and ζ‐potential

The preparation of SNEs was based on a self‐emulsifying system, and Ph was solubilized in the oily core of the nanoemulsion structures with F127& P123 mixture coating (Figure 2). The optimized compositions of SNEs are listed in Table 1. The particle size, polydispersity index (PDI), and zeta potential of different SNEs and the SNEs during 30 days storage are shown in Table 2. All values of particle size, PDI and zeta potential are not obviously increased, indicating the SNEs are still fairly stable during 30 days storage.

**TABLE 2:** The size, size distribution, and ζ‐potential of the Initial SNEs and SNEs after 30 days

Formulation	Initial SNEs			SNEs after 30 days		
	Mean particle size (nm)	Polydispersity index (PDI)	ζ‐potential (mV)	Mean particle size (nm)	Polydispersity index (PDI)	ζ‐potential (mV)
SCT‐Ph‐SNE	29.73 ± 1.30	0.268 ± 0.035	−3.4 ± 1.9	31.12 ± 2.20	0.298 ± 0.029	−3.1 ± 1.5
MCT‐Ph‐SNE	37.13 ± 3.51	0.202 ± 0.026	−2.8 ± 1.8	39.28 ± 2.41	0.240 ± 0.010	−2.5 ± 1.2
LCT‐Ph‐SNE	48.96 ± 2.96	0.189 ± 0.023	−2.1 ± 1.6	52.01 ± 1.16	0.221 ± 0.030	−2.1 ± 1.8

Note

Values are mean ± standard deviation (*n* = 3).

**FIGURE 3:**
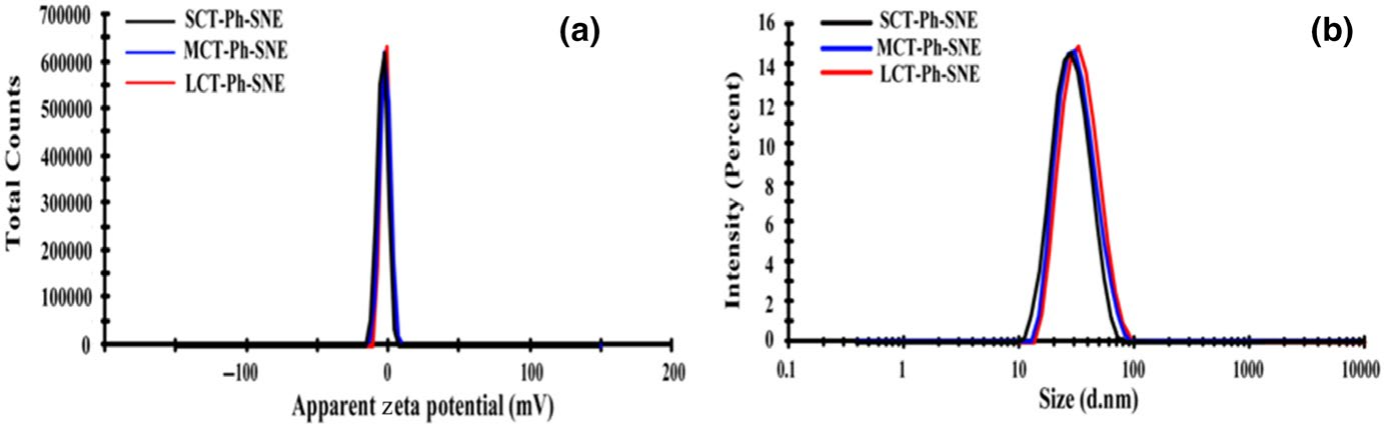
(a) Size distribution (nm) and (b) surface zeta potentials (mV) of SNE formulations in the water

The size of LCT‐Ph‐SNE, MCT‐Ph‐SNE, and SCT‐Ph‐SNE containing Ph (4%w/w) is 29.73 ± 1.30 nm, 37.13 ± 3.51 nm, and 48.96 ± 2.96 nm. Only a slight increase in the particle size was observed in different SNEs (Figure 3). The SNEs had an average size of approximately 38.60 nm. It is evident that smaller nanoparticle could significantly promote penetration and transport through the mucus layer (Wilde et al., 2019). Therefore, this small droplet size is considered ideal to result in enhanced absorption and bioavailability through intestinal mucosal surfaces.

In previous work that the size and zeta potentials of SNEs without modification were around −20.0 mV (Xia et al., 2017). In this study, the zeta potentials of SNEs were in the range of −3.4 to −2.10 mV (close to neutral). The increase of zeta potential showed that the extension of the hydrophilic polyethylene oxide (PEO) chains of P123/F127 mixture coated the oil phase surface, which had further increased the hydrophilicity of SNEs and shielding of droplets’ surface charges. Moreover, PEG‐modified nanoparticles with neutral surface charges undergo more rapid transport than those anionic charges in mucus (Lai, Wang, & Hanes, 2009). Therefore, we estimated that this optimized SNEs with near‐neutral charge would appear rapid transport and then highly absorption by oral administration.

Ethanol and 1,2‐Propanediol: Water phase and solvent. It can improve the solubility of Phloretin. Medium‐chain triglycerides, Glyceryl tributyrate and MAISINE® 35‐1: Oil phase Cremophor EL: Nonionic surfactants. Pluronic® P123 and Pluronic® F127: Triblock copolymer surfactants modify self‐nanoemulsions. Its hydrophilic long chain can greatly enhance the diffusion ability of phloretin in mucus.

#### 3.1.2 TEM morphology analysis

**FIGURE 4:**
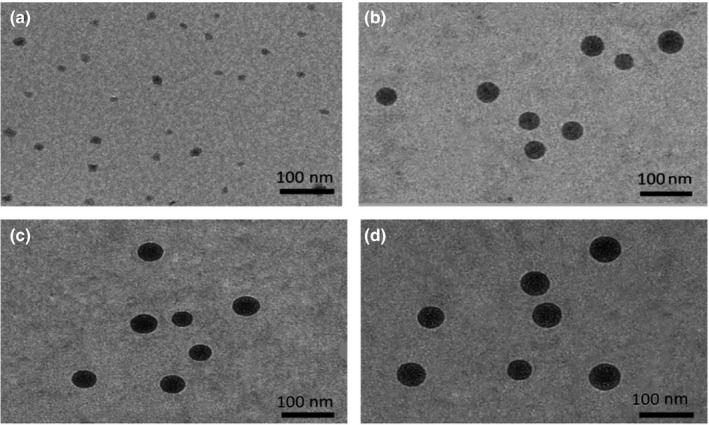
TEM photographs of (a) SNE without mixed polymeric modified, (b) SCT‐Ph‐SNE, (c) MCT‐Ph‐SNE and (d) LCT‐Ph‐SNE after 24 hr postdilution in distilled water. Data are mean ± *SD* (*n* = 3)

**FIGURE 5:**
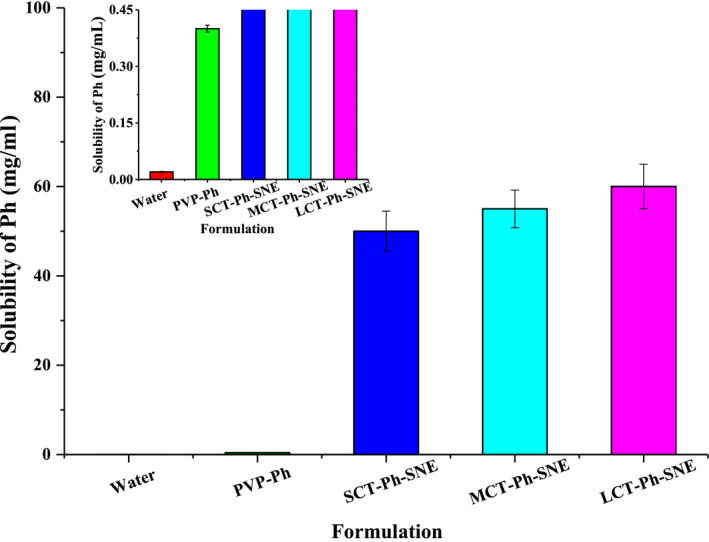
The solubility of different SNEs formulation (PVP‐Ph, SCT‐Ph‐SNE, MCT‐Ph‐SNE, and LCT‐Ph‐SNE) compared with Ph. Data are shown as the means ± standard deviations (SDs). (*n* = 5)

As shown in Figure 4, the morphology of SNEs observed by a transmission electron microscope (TEM) after 24 hr in distilled water. All nanoemulsions (Figure 4b‐d) loaded Ph were less than 100 nm in size and the spherical nature in shape. That is almost consistent with that obtained in the droplet size analysis in Table 2. Furthermore, the clear spherical shell without the sign of Ph precipitation was observed in the images (Figure 4b‐d), indicating that Ph was encapsulated into the SNEs and stability of the formed nanoemulsions. However, the shape of the SNE without mixed polymeric modified is similarly oval (Figures 4a and 5).

### 3.2 Physical properties

#### 3.2.1 Solubility study, interfacial tension, and apparent viscosity

**FIGURE 6:**
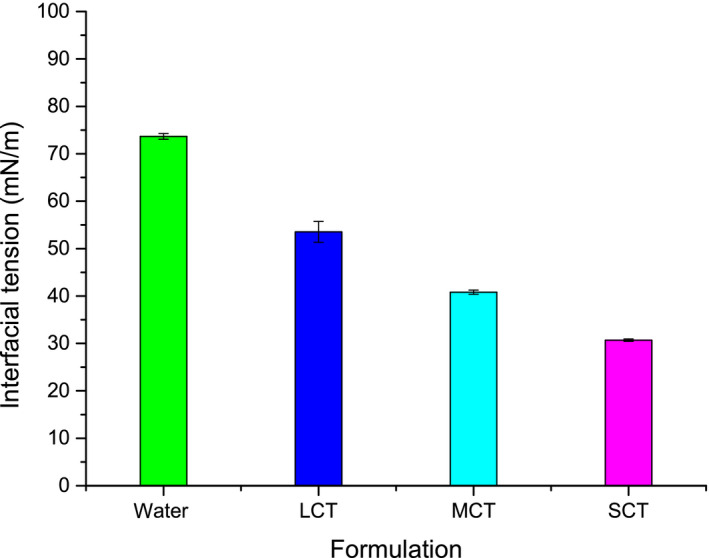
Interfacial tension (mN/m) for various SNE formulations (SCT‐Ph‐SNE, MCT‐Ph‐SNE and LCT‐Ph‐SNE). Data are shown as the means ± standard deviations (SDs). (*n* = 5)

The result of the solubility study of Ph in various SNEs has been shown in Figure 5. The solubility of Ph in SCT‐Ph‐SNE, MCT‐Ph‐SNE, and LCT‐Ph‐SNE was 50 ± 4.5, 55 ± 4.2, 60 ± 5.0 mg/ml, respectively. The Figure 6 illustrated that there was a small increase in solubility of Ph by longer fatty acid chain length in the SNE formulations in vitro solubility of Ph at room temperature. However, comparing with the solubility of Ph solution (approximately 0.02 mg/ml), the solubility of LCT‐Ph‐SNE had a significant increase up to 3,000‐fold. The solubility study of SNEs was found to be in the following order: Ph solution < PVP‐Ph < SCT‐Ph‐SNE < MCT‐Ph‐SNE < LCT‐Ph‐SNE. It also showed that hydrophobic drugs had high solubility in lipid‐based formulations (Heshmati, Cheng, Eisenbrand, & Fricker, 2013).

Based on the results reported in Figure 6, we fund that the interfacial tension of SNEs was in the following order: SCT‐Ph‐SNE (30.7 mN/m) < MCT‐Ph‐SNE (40.8 mN/m) < LCT‐Ph‐SNE (53.5 mN/m) < water (73.7 mN/m). Obviously, compared with water, the interfacial tension of SNEs decreased steeply from 73.7 to 30.6 mN/m. In general, the surfactants could decrease the particle size and have an impact on the interfacial tension and apparent viscosity (McClements & Rao, 2011). As anticipated, the F127/P123 polymeric mixture had high mobility at the interface, which significantly decreased the surface tension and promoting droplets’ disruption. On the other hand, though the SNEs stabilized by the same interfacial surfactant layer, the interfacial tension increased with longer length of fatty acid chain in the oils.

**FIGURE 7:**
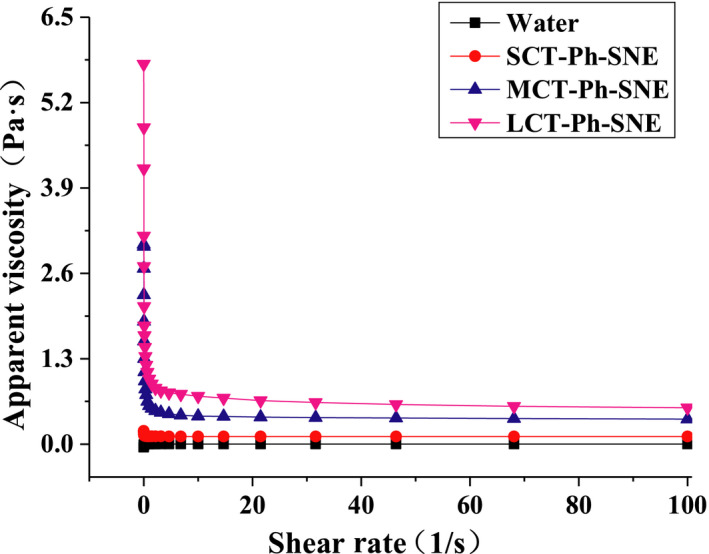
Apparent viscosity of different SNEs formulation (SCT‐Ph‐SNE, MCT‐Ph‐SNE and LCT‐Ph‐SNE) from 0 to 100 s^−1^ shear rate

Moreover, the surfactants not only influenced the interfacial tension, but also the apparent viscosity. The rheological properties of the SNEs were studied (Figure 7). With increasing in shear rate, the viscosity of emulsions decreased due to typical shear thinning behavior. The viscosity of SNEs in following order: Water (approximately 0.0) < SCT‐Ph‐SNE (from 0.20 ± 0.05 to 0.12 ± 0.01 Pa·s) < MCT‐Ph‐SNE (from 3.04 ± 0.15 to 0.38 ± 0.02 Pa·s) < LCT‐Ph‐SNE (from 5.79 ± 0.05 to 0.55 ± 0.11 Pa·s). As previously reported, the emulsions were more physically stable in terms of smaller droplet size and higher viscosity (Yesiltas et al., 2018). Due to the long chain in the oil phase, the SNEs with higher apparent viscosity improved physically stable.

#### 3.2.2 In vitro stable study

**FIGURE 8:**
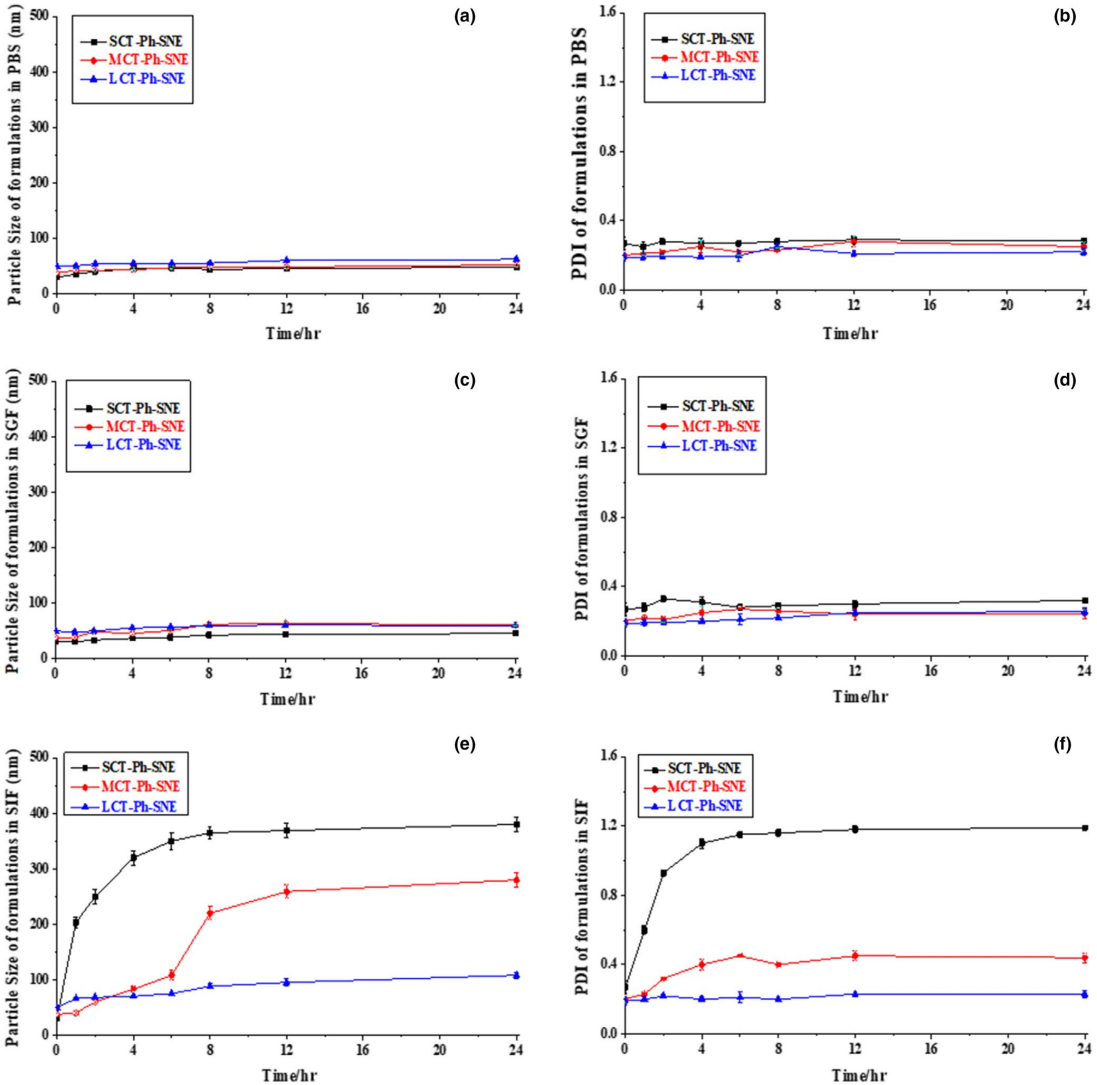
The particle size and PDI of SNEs in phosphate‐buffered saline (PBS) (a‐b), simulated gastric fluid (SGF) (c‐d), and simulated intestinal fluid (SIF) (e‐f)

Before conducting in vivo studies, it was necessary to ensure the stability of SNE nanoparticles. In this study, we used all kinds of buffers and biorelevant medias such as PBS, SGF, and SIF to investigate the stability of SNEs. As shown in Figure 8a‐d, there were no significant changes in particle size and PDI observed in PBS and SGF buffers in 24 hr. This suggested that the structure of SNEs particles did not undergo obviously damaged; thus, it could make more resistance to the acidic conditions in the intestinal fluid (SIF). Nevertheless, particle size and PDI of SCT‐Ph‐SNE had increased dramatically in SIF (Figure 8e‐f), which meant that the structure of particles had damaged and then the mixed F127/P123 polymeric membrane had been broken down. The SCT‐Ph‐SNE digested much easier than other SNEs by lipase in the intestinal environment, which was likely because of the short‐chain oil. Hereby, it showed that the digestion rate was relying on the length of the fatty acid chains, the longer the fatty acid chains, the more efficient in delaying the digestion rate. Finally, we found the LCT‐Ph‐SNE was the most stable in vitro stable study.

Based on the above results, the SNEs were selected as a study subject to evaluate its pharmacokinetic study and bioefficacy study.

### 3.3 In vitro lipolysis study

**FIGURE 9:**
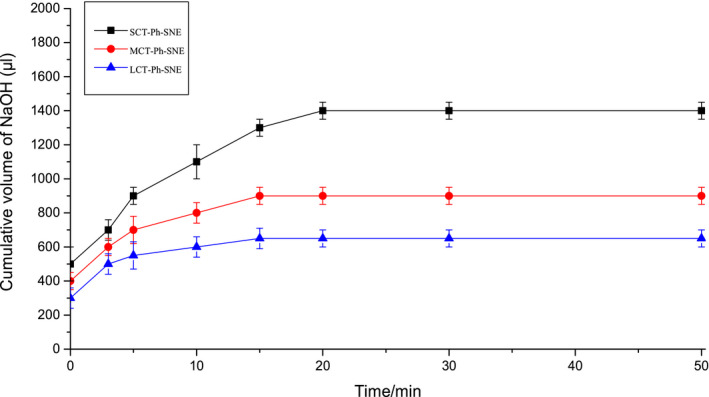
In vitro digestion curve for SCT‐Ph‐SNE, MCT‐Ph‐SNE, and LCT‐Ph‐SNE. Each system contained 100 mg SNEs (*n* = 3)

To simulate in vivo lipid biodegradation, in vitro lipolysis study commonly used by the alkaline compensation method (Han et al., 2009) though it is just an accelerated process. As shown in this study (Figure 9), all SNEs decorated with F127/P123 polymeric mixture can be completely degraded in vitro within 20 min. Comparing various SNEs, we could found that SCT‐Ph‐SNEs were the most rapid degradation rate, while LCT‐Ph‐SNE digested slowest owing to the long fatty acid chain in the oil. The results indicated that the LCT‐F127‐SNE could have a better capacity for resisting digestion than other SNEs. Also, the longer the fatty acid chains of SNEs, the slower the digestion rate. As previously reported that smaller nanoemulsions contributed to faster digestion due to their significantly extended specific surface area (Tan, Colliat‐Dangus, Whitby, & Prestidge, 2014). As shown in Supplementary Table 1, the Ph was all released from SNEs and then distributed between the aqueous phase and the precipitate phase after 40 min digestion by using an in vitro lipid digestion model. Approximately 24.6%, 16.6%, and 4.2% of drug (Ph) precipitated from SCT‐Ph‐SNE, MCT‐Ph‐SNE, and LCT‐Ph‐SNE, respectively. The LCT‐Ph‐SNE (96.6% in digestion) was not completely lipolyzed after 40 min digestion, compared with the SCT‐Ph‐SNE and MCT‐Ph‐SNE. The least drug precipitation from LCT‐Ph‐SNE illustrated that the LCT‐Ph‐SNE was highly effective in inhibiting lipid digestion.

### 3.4 Pharmacokinetic study

**FIGURE 10:**
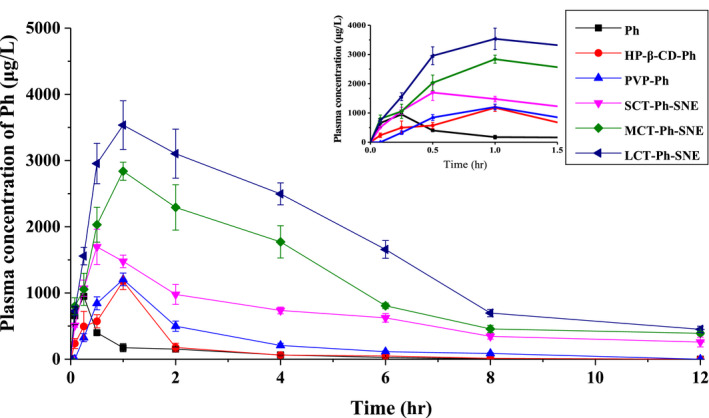
Plasma concentration versus time profile of different formulations after oral administration in a dose equivalent to 400 mg/kg. The small chart was magnified. Data are shown as the means ± standard deviations (SDs). (*n* = 5)

The principal goal of oral drug delivery is to improve the bioavailability of poorly water‐soluble agents. For this purpose, plasma pharmacokinetics were evaluated after oral administration (400 mg·kg^−1^) in the rat. Plasma samples of Ph solution, HP‐β‐CD‐Ph (solution), PVP‐Ph (suspension), SCT‐Ph‐SNE, MCT‐Ph‐SNE, and LCT‐Ph‐SNE were analyzed at preset time points. As shown in Figure 10, after oral administration of different samples, the plasma concentration of Ph gradually increased within 1 hr (*C*_max_) and then declined within 12 hr. The pharmacokinetic parameters are shown in Table 3.

**TABLE 3:** Pharmacokinetic (PK) parameters of Ph following oral administration of Ph suspensions and SNEs formations in rates

Formulation	*C*_max_ (μg/L)	*T*_max_ (h)	AUC_0−24_ (h·μg/L)	*F* (%)
Ph	1,149.26 ± 11.26	0.25	3,017.65 ± 476.65	100.00%
HP‐β‐CD‐Ph	1,177.43 ± 124.86***	0.50	3,906.65 ± 869.48***	129.46%
PVP‐Ph	1,204.70 ± 95.43***	0.50	4,819.82 ± 366.76***	159.72%
SCT‐Ph‐SNE	1796.75 ± 193.68***	1.00	11,469.10 ± 1,239.35***	380.07%
MCT‐Ph‐SNE	2,838.70 ± 136.07***	1.00	17,018.83 ± 2025.41***	563.98%
LCT‐Ph‐SNE	3,534.90 ± 367.36***	1.00	23,703.54 ± 2,276.77***	785.50%

Note

*C*_max_: maximum plasma concentration; *T*_max_: time at *C*_max_; AUC0‐24 hr, area under the concentration‐time curve from time 0–24 hr; *F*: relative oral bioavailability (%) = [AUC (test)/AUC (control)] × [Dose(control)/Dose (test)] × 100% (Nazari‐Vanani, Moezi, & Heli, 2017). Data are shown as the means ± standard deviations (SDs). (*n* = 5).

*** *p* < .001 compared with the Ph group.

The AUC_0‐24 hr_ and C_max_ of all SNE formulations were much higher than that of the Ph solution, HP‐β‐CD‐Ph and PVP‐Ph (Ph suspension). The AUC_0‐24 hr_ of Ph from the LCT‐Ph‐SNE was significantly greater than that from Ph solution (7.9‐fold), HP‐β‐CD‐Ph (6.0‐fold), and PVP‐Ph (4.9‐fold), respectively. Similarly, the LCT‐Ph‐SNE had the best *C*_max_ in all samples. The *T*_max_ comparison suggests that the SNE formulations took a longer time (1.5 hr) to reach *C*_max_ compared with Ph solution (0.25 hr). The Ph solution concentration in plasma decreases abruptly, but the self‐nanoemulsions system could help to postpone the release of Ph. Rapidly distributed and quickly metabolized of Ph results in less *T*_1/2_. Additionally, we found that the Ph aqueous suspension displayed low drug bioavailability due to the low aqueous solubility of Ph and then the Ph would be easy to precipitate from the suspension. Take together the in vitro data, we could observe that the oral bioavailability is closely interrelated to the stability and physical properties of SNEs such as solubility study, interfacial tension, apparent viscosity and so on. That is consistent with previous researches about the emulsion (Arshad & Andreas, 2018; Yuting et al., 2017).

Overall, we observed that the optimizing SNEs system was quite effective to enhance oral bioavailability, compared with other carriers (HP‐β‐CD‐Ph, PVP‐Ph). And the oral bioavailability of HP‐β‐CD‐Ph is just a little better than that of the PVP‐Ph. Among these samples, the oral bioavailability is in the order PVP‐Ph > HP‐β‐CD‐Ph**»** SCT‐Ph‐SNE > MCT‐Ph‐SNE > LCT‐Ph‐SNE. And the increase in bioavailability of the SNEs system is more likely to inhibit digestion by enzyme and promoted mucus penetration, which led to high oral bioavailability (Song et al., 2018). It could be used as a possible formulation for poorly water‐soluble agents to improve its oral bioavailability.

### 3.5 Anti‐inflammatory effect

**FIGURE 11:**
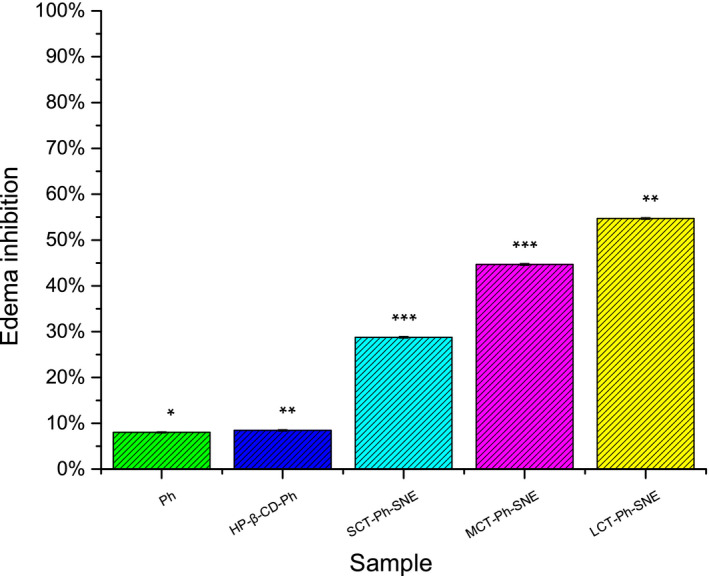
The edema inhibition rates of Ph solution and nanoemulsions ( SCT‐Ph‐SNE, MCT‐Ph‐SNE and LCT‐Ph‐SNE); *p* ≤ .05 (⁎), *p* ≤ .01 (⁎⁎) and *p* < .001(⁎⁎⁎)

To further prove that the above‐mentioned data were good estimations in bioefficacy of the SNEs system, we observed the anti‐inflammatory effect of optimized SNEs compared with Ph solution after oral administration in a dose equivalent to 200 mg/kg. Lots of literature demonstrated that phloretin had excellent anti‐inflammatory in various inflammation diseases (Kim et al., 2014; Song et al., 2018; Wei et al., 2017). The experimental evidence has shown that exposure of skin to 12‐O‐tetradecanoyl‐phorbol acetate (TPA). TPA would induce a pleiotropic tissue response encompassing a strong inflammatory reaction (Adami et al., 2012; Oliveira et al., 2017). Then, the anti‐inflammatory effect was evaluated in a TPA‐induced rat ear edema model. It has been proved that increased skin edema is the first hallmark of skin inflammation including the process of increased vascular permeability and proliferation of epidermal keratinocytes (Nickoloff, Ben‐Neriah, & Pikarsky, 2005). It can be seen from Figure 11 that TPA topical treatment alone results in a rat ear erythema and edema, and then, the average weight of TPA‐treated ear punches increased from 8.0 to 22.5 mg, increased by 281% after 6 hr. The oral administration of all SNE formulations could potently suppress TPA‐induced ear swelling compared with Ph solution. Oral treatment of Ph solution caused an 8.0% decrease in the weight of the ear punches, which is similar to that of HP‐β‐CD‐Ph (8.5%). The suppression rates of SCT‐Ph‐SNE and MCT‐Ph‐SNE were 28.8% and 44.7%, respectively. Notably, LCT‐Ph‐SNE topical pretreatment resulted in a decrease of 54.7%, which is about 6.8‐fold than that of Ph solution. As the previous result in pharmacokinetic study, the anti‐inflammatory activity of the Ph and HP‐β‐CD‐Ph was very poor effective than that of the SNEs. And the most obvious enhancement of anti‐inflammatory effect is the LCT‐Ph‐SNE compared with three kinds of SNEs. The reason should be due to the more oral bioavailable through the SNEs system in pharmacokinetic study. Therefore, we continue studying for these SNEs by the below histological analysis.

**FIGURE 12:**
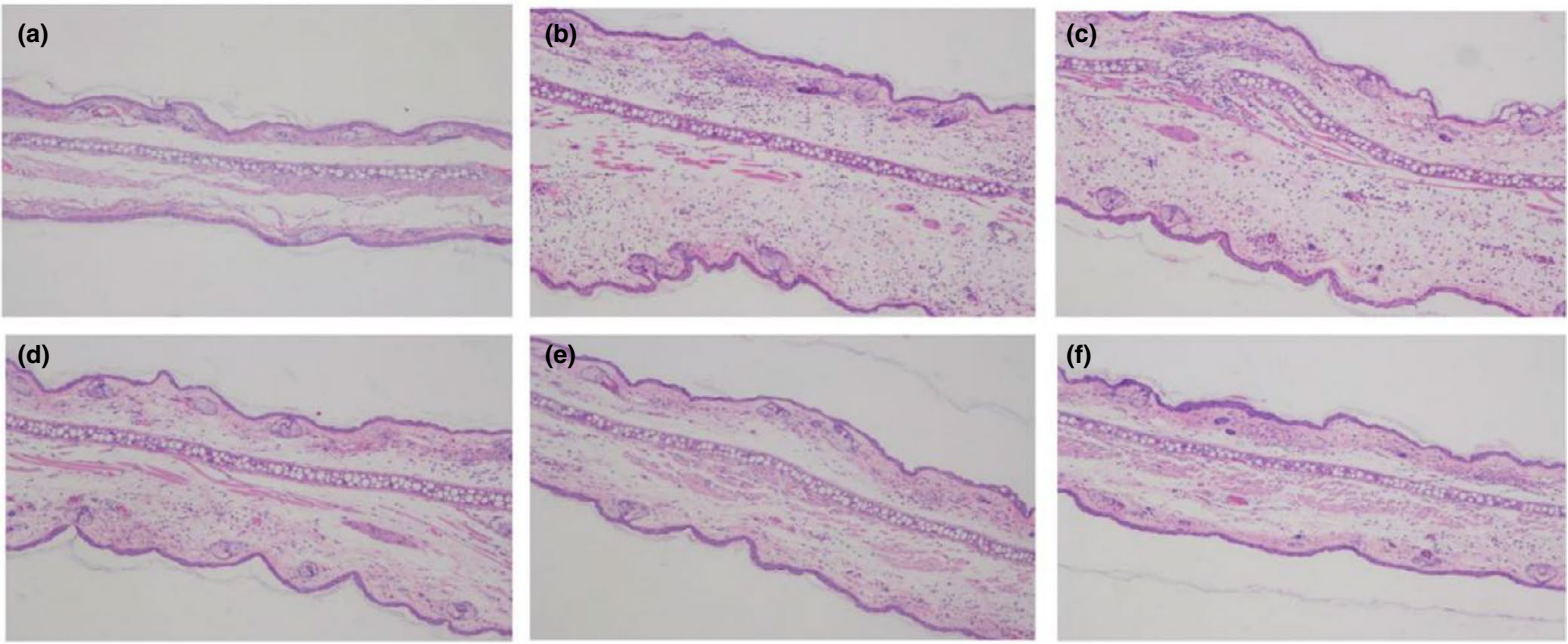
The anti‐inflammatory effects of nanoemulsions on histopathological changes stained with H&E stain. (a) Normal group (treated with water); (b) TPA（100 μg/ml); (c) Ph; (d) SCT‐Ph‐SNE; (e) MCT‐Ph‐SNE; (f) LCT‐Ph‐SNE. Data are expressed as mean ± S.D. (*n* = 5). Magnification 200×

Histological appearance evaluation has always been the appropriate way to assess the disorders of inflammatory tissue (Liu, Li, Zheng, Zhang, & Du, 2015). Then, we observed the effect of the Ph solution and the SNEs on histological appearances of rat ear. The histological appearances of the ear sections showed in Figure 12a that the normal group treated with water displayed the normal appearance in the epidermal layer without any obvious lesion. In contrast, the TPA topical treatment caused an obvious inflammation response with clear evidence of ear edema and inflammatory cell infiltration (Figure 12b). As the suppression rate above, all SNEs could significantly suppress the signs of inflammatory responses (Figure 12d–f). Nevertheless, in comparison with the inhibitory effects of these SNE formulations, the LCT‐Ph‐SNE showed the most decreased inflammatory cell infiltration and its effect is better than that of SCT‐Ph‐SNE and MCT‐Ph‐SNE (Figure 12f).

**FIGURE 13:**
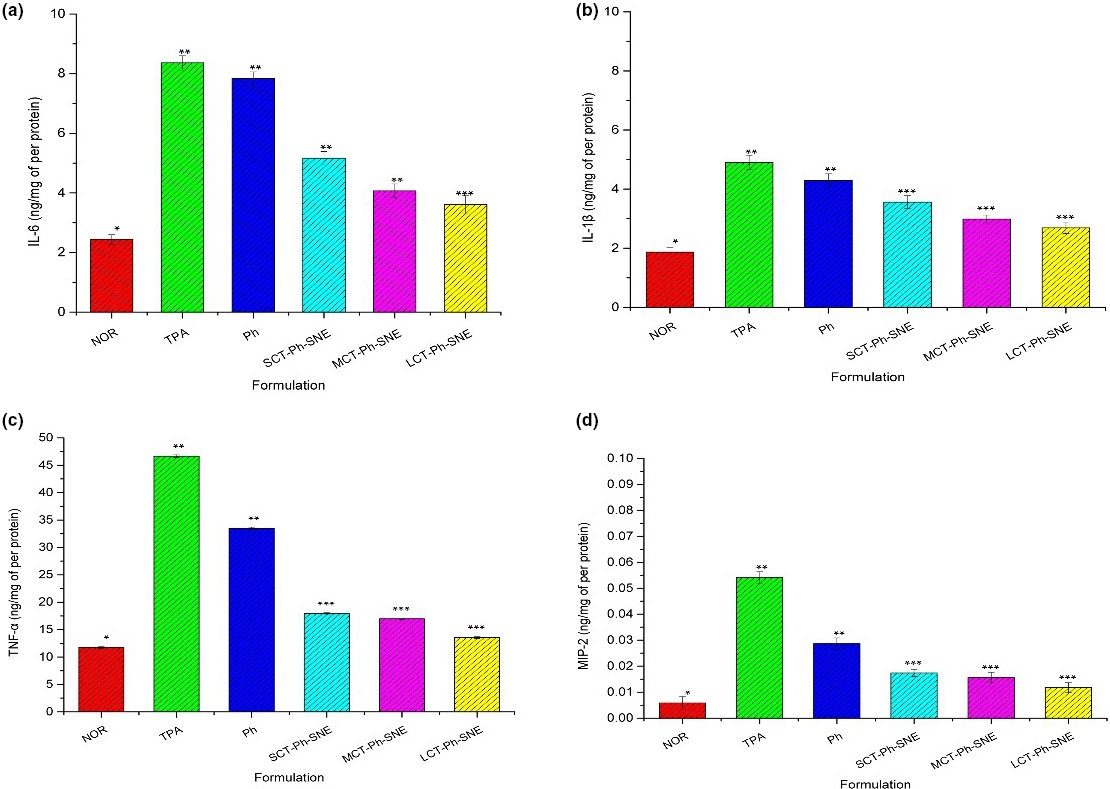
The proinflammatory mediators (IL‐6, IL‐1β, TNF‐α and MIP‐2) were measured 6 hr in mouse ears. (*n* = 5), NOR: Normal group (treated without TPA); TPA group（100 μg/ml); Ph group (Ph + TPA); SCT‐Ph‐SNE group (SCT‐Ph‐SNE + TPA); MCT‐Ph‐SNE group (MCT‐Ph‐SNE + TPA); LCT‐Ph‐SNE group (LCT‐Ph‐SNE + TPA); *p* ≤ .05 (⁎), *p* ≤ .01 (⁎⁎) and *p* < .001(⁎⁎⁎)

As we known, the proinflammatory mediators (IL‐6, IL‐1β, TNF‐α, and MIP‐2) play a significant role in the inhibition of inflammation and cartilage destruction, which could slow down the arthritis progression (Liu et al., 2015). The Figure 13 showed that the SNEs formulations had significantly diminished the proinflammatory mediators of IL‐6, IL‐1β, TNF‐α, and MIP‐2 generation in the ears compared with the TPA group and Ph group, indicating that SNEs may exert an anti‐inflammatory effect by suppressing macrophage function. Among these proinflammatory mediators, the TNF‐α values are the highest and MIP‐2 values are the lowest. The anti‐inflammatory effect of the SNEs was found to be in the following order: Ph « SCT‐Ph‐SNE < MCT‐Ph‐SNE < LCT‐Ph‐SNE.

Taken together with data of inhibition on ear edema and proinflammatory mediators, these results specify the best anti‐inflammatory of LCT‐Ph‐SNE compared with the unformulated Ph. Additionally, in all SNEs samples, the LCT‐Ph‐SNE has the best anti‐inflammatory activity, which may be a potential treatment of inflammation‐associated diseases such as rheumatoid arthritis. And it should deserve more investigation because it may be a beneficial food for further development in clinical application.

## 4 CONCLUSION

In this study, we reasonably designed and investigated multifunctional SNE formulations for the oral delivery of hydrophobic components. The LCT‐Ph‐SNE had nearly neutral surface charge and better stability in different biorelevant media. The presence of a mixed hydrophilic group (F127/P123) on the surface of the oil droplet generated a steric hindrance, which is more likely to reduce enzymatic degradation in the gastrointestinal tract. Furthermore, efficient oral absorption of Ph was achieved by the LCT‐Ph‐SNE, with 6.8‐fold higher AUC0‐24 hr values compared with those of Ph. Overall, we demonstrated that our SNEs were able to effectively enhance the oral bioavailability and bioefficacy of hydrophobic drug phloretin by the SNEs, while the mucus‐penetrating ability of this formula needs further investigations based on current results. Also, this novel SNE formulation may be used for other poorly soluble drugs, which will be a promising platform for oral delivery.

## CONFLICT OF INTEREST

The authors declare no conflicts of interest.

## Supporting information

Supplementary Material
