# Supplementary material for: Enhanced oral bioavailability and bioefficacy of phloretin using mixed polymeric modified self‐nanoemulsions
Source: Food Sci Nutr. 2020 May 28;8(7):3545–58. doi: 10.1002/fsn3.1637 (PMC7382203; doi:10.1002/fsn3.1637)
Supplement: Supplementary file 1 — Supplementary Material [file FSN3-8-3545-s001.docx]

**Supplementary Information**


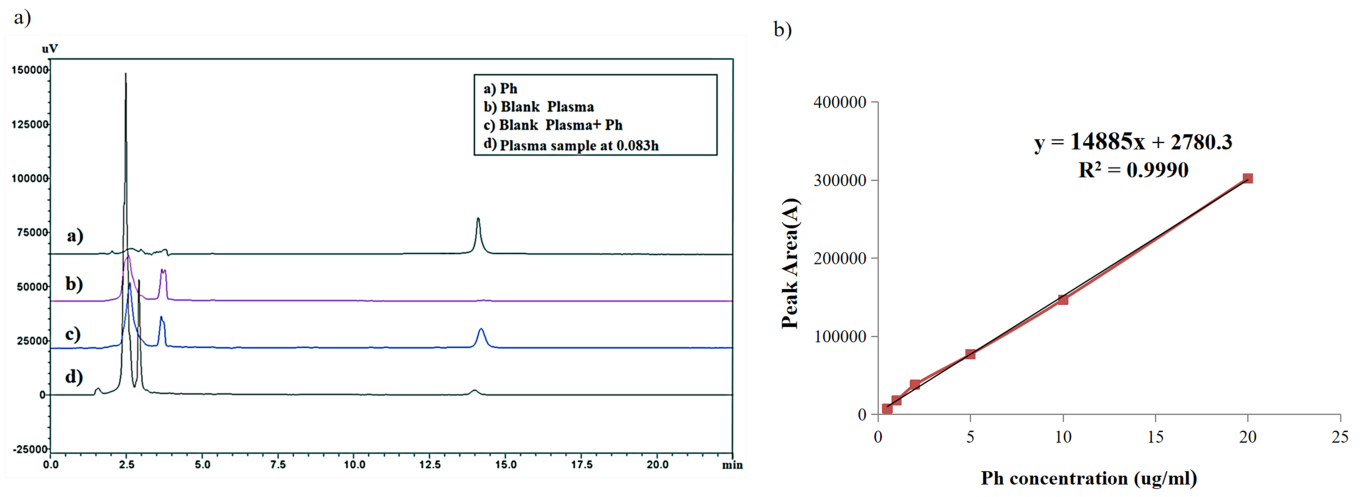


**Supplementary Figure S1**. a) HPLC chromatographic of the plasma sample. b) The standard curve of Ph plasma concentration. Data are shown as the means±standard deviations (SDs). (n=5)

**Supplementary Table 1**. HPLC analysis of phloretin (Ph) in vitro lipolysis study

| **Parameter** | **SCT-Ph-SNE** | **MCT-Ph-SNE** | **LCT-Ph-SNE** |
| --- | --- | --- | --- |
| % in aqueous phase | 75.4 | 83.4 | 95.8 |
| % in precipitate | 24.6 | 16.6 | 4.2 |
| % in oil | 0.0 | 0.0 | 0.0 |
| % in degestion | ~99.9 | ~98.5 | ~96.6 |

The concentration of phloretin (Ph) after 40 min digestion of 100mg LCT, MCT or SCT from SNEs by using an in vitro lipid digestion model (n=3). % in degestion= Vt / V100%, V100%：the cumulative volume of NaOH versus the time curve reached the stagnation phase without the oil phase after centrifugation.Vt: the cumulative volume of NaOH used at one time point.
